# Deep-learning denoising for ultrahigh-resolution photon-counting detector CT: phantom and in vivo evaluation of non-calcified coronary plaques

**DOI:** 10.1007/s10554-026-03774-x

**Published:** 2026-07-10

**Authors:** Sardi Hyska, Muhammad Taha Hagar, José Osoria-Velasquez, Nicola Fink, Jens Ricke, Moritz C. Halfmann, Hugo Marques, Elliot McVeigh, George Wesbey, Pál Maurovich-Horvat, Bálint Szilveszter, Tilman Emrich, Akos Varga-Szemes, Milan Vecsey-Nagy

**Affiliations:** 1 Department of Radiology and Radiological Science, Medical University of South Carolina, 25 Courtenay Dr, Charleston, SC 29425 USA; 2 Department of Radiology, LMU University Hospital, LMU Munich, 15 Marchioninistraße, 81377 Munich, Germany; 3 Department of Diagnostic and Interventional Radiology, Medical Center, Faculty of Medicine, University of Freiburg, University of Freiburg, 55 Hugstetter Straße, 79106 Freiburg, Germany; 4 Department of Diagnostic and Interventional Radiology, University Medical Center of the Johannes Gutenberg-University, 1 Langenbeckstraße, 55131 Mainz, Germany; 5 UNICA Radiology, MRI unit Hospital da Luz Lisboa, Católica Medical School, 100 Av. Lusíada, Cardiovascular, Lisboa, 1500-650 CT Portugal; 6 Department of Medicine, UC San Diego School of Medicine, 9500 Gilman Dr, La Jolla, 92093 CA USA; 7 Department of Cardiovascular Division, UC San Diego School of Medicine, 9500 Gilman Dr, La Jolla, 92093 CA USA; 8 Department of Radiology, UC San Diego School of Medicine, 9500 Gilman Dr, La Jolla, 92093 CA USA; 9 Department of Bioengineering, UC San Diego School of Medicine, 9500 Gilman Dr, La Jolla, 92093 CA USA; 10 Department of Cardiology and Radiology, Scripps Clinic, 9888 Genesee Ave, La Jolla, 92037 CA USA; 11 Department of Radiology, Medical Imaging Center, Semmelweis University, 2 Koranyi Sandor street, Budapest, 1083 Hungary; 12 Heart and Vascular Centre, Semmelweis University, 68 Varosmajor street, Budapest, 1122 Hungary; 13 German Centre for Cardiovascular Research, Partner Site Rhine-Main, 1 Langenbeckstraße, 55131 Mainz, Germany

**Keywords:** coronary artery disease, coronary computed tomography angiography, photon-counting detector, non-calcified plaque, ultrahigh-resolution

## Abstract

To assess the value of convolutional neural network (CNN)-based denoising for the evaluation of non-calcified coronary plaques on ultrahigh-resolution (UHR) photon-counting detector (PCD) coronary CT angiography (CCTA). A dynamic phantom containing lipid-rich and fibrotic plaques with 50%-diameter stenosis (PDS) was scanned on PCD-CT under varying conditions. For in-vivo imaging, consecutive patients with non-calcified coronary plaques (NCPs) who underwent CCTA with PCD-CT were included. Image series were reconstructed using a sharp vascular kernel (Bv64) with slice thicknesses of 0.2 mm/0.4 mm, quantum iterative reconstruction (QIR) levels 3/4, and with/without CNN denoising. Phantom-based line-profile analysis yielded edge-width at half maximum (EWHM) and 10–90% rise distance as sharpness metrics. Plaque contrast-to-noise ratio (CNR) and PDS were assessed in the phantom and in patients. Two readers evaluated subjective image quality (noise, diagnostic confidence) using a four-point Likert scale. Pairwise comparisons were performed using a Bonferroni-corrected *p* < 0.002 for significance. Fifty-five patients (median age 74 [66.5–78] years; 19 women) with 97 NCPs were included. Phantom-based sharpness metrics remained unchanged after denoising (all pairwise *p* > 0.051). CNN denoising increased plaque CNR consistently in the phantom (fibrotic: 0.80–1.64 to 0.92–2.06; lipid-rich: 0.21–0.34 to 0.35–0.46) and in vivo (0.99–2.07 to 1.37–2.92, all pairwise *p* < 0.001). Denoising did not alter PDS values in phantom or in vivo plaques (all pairwise *p* > 0.006). Subjective image noise and diagnostic confidence improved across all reconstructions (all pairwise *p* < 0.001). CNN-based denoising of UHR PCD-CT improves image quality of non-calcified coronary plaques, while preserving sharpness and quantitative stenosis metrics.

**Supplementary Information:**

The online version contains supplementary material available at https://doi.org/10.1007/s10554-026-03774-x.

## Introduction

The first clinical photon-counting detector (PCD)-CT represents a major advance in cardiac imaging by offering ultrahigh-resolution (UHR) coronary CT angiography (CCTA) with substantially reduced partial-volume effects around high-density structures [1, 2]. As a result, optimization efforts have primarily focused on calcified plaques, where reconstruction settings designed to maximize spatial resolution provide clear benefits in plaque delineation [3]. However, these UHR strategies inherently increase image noise, a trade-off that is acceptable for calcified plaques due to the magnitude of blooming reduction but may not benefit the visualization of non-calcified plaques (NCPs) to the same extent [4]. NCPs, particularly low-attenuation plaques, represent the morphological substrate of plaque vulnerability and are associated with major adverse cardiovascular events [5, 6]. However, the visualization of NCPs remains challenging, as noise degrades low-contrast texture and edge definition, and conventional iterative reconstruction methods may introduce spatial smoothing or alter image texture, potentially obscuring plaque features [7, 8]. Convolutional neural network (CNN)-based denoising represents a complementary strategy that may mitigate noise while preserving attenuation and anatomical detail. While feasibility has been demonstrated in cardiac CT, its role in UHR PCD-CT for NCP evaluation remains insufficiently characterized [9, 10].

The aim of this study was to assess the added value of post-hoc denoising with a commercially available U-Net–based CNN for the evaluation of non-calcified coronary plaques on UHR PCD-CCTA, using combined phantom and in vivo datasets. We hypothesized that CNN denoising would improve image quality of NCPs without compromising sharpness, quantitative stenosis measurements, or diagnostic confidence.

## Materials and methods

### Phantom model

A chest phantom (Cardio CT Phantom; QRM) simulating a medium-sized patient was employed [11]. The mediastinal space contained a water tank with three artificial coronaries (outer diameter of 4.5 mm, inner diameter 3 mm). Each vessel included two NCPs, one fibrotic (100 Hounsfield units [HU] on energy-integrating detector [EID]-CT at 120 kVp) and one lipid-rich (-25 HU on EID-CT at 120 kVp), each forming a 50% diameter stenosis (PDS). The vessels were filled with blood-contrast mixtures, producing attenuations of 300 HU, 400 HU, and 500 HU (EID-CT at 120 kVp). Coronary motion was simulated in four dimensions (Sim4DCardio; QRM), enabling static and dynamic acquisitions at different heart rates.

### Study population

The study was approved by the institutional review boards and conducted in accordance with HIPAA regulations and the Declaration of Helsinki (approval number: Pro00131646). Consecutive patients aged 18 years or older who underwent clinically indicated UHR CCTA for evaluation of stable chest pain or as part of transcatheter aortic valve replacement (TAVR) workup were retrospectively included. Exclusion criteria were the lack of raw CT projection data and the absence of non-calcified coronary plaques.

### CT acquisition

All scans were performed on a first-generation dual-source PCD-CT system (NAEOTOM Alpha, Siemens Healthineers) in UHR mode (120 × 0.2 mm collimation). The phantom was scanned statically and at 60 and 85 beats per minute (bpm) with three repetitions per acquisition. Scan parameters included a tube potential of 120 kVp and an effective mAs of 98. Patients were scanned using tube potentials 90–140 kVp and image quality (IQ) levels 50–75 in either a sequential or spiral acquisition mode. Patients received 60–90 mL of iopromide (Ultravist 370, Bayer Healthcare), or iomeprol (Iomeron 400, Bracco Diagnostics) contrast material at flow rates of 3.5–5.0 mL/s. Stable chest pain patients were administered sublingual (0.4 mg) nitroglycerin and, if the heart rate was higher than 75 bpm, intravenous beta-blocker (5 mg).

### CT image reconstruction and denoising

For each UHR scan, four datasets were generated by combining two slice thicknesses (0.2 mm and 0.4 mm) and quantum iterative reconstruction (QIR) levels (3 and 4), using the sharp vascular kernel optimized for UHR imaging (Bv64) [3]. Denoised datasets were generated for all reconstructions using a commercially available CNN-based on a U-Net architecture (ClariCT.AI, version 1.0, ClariPi). The algorithm was originally trained on large-scale EID-CT datasets and subsequently adapted for PCD-CT [9, 12, 13].

### Edge sharpness measurements

Plaque edge sharpness in the phantom model was assessed using line profile analysis in the open-source software FIJI (ImageJ version 1.54p, Wayne Rasband, National Institutes of Health, USA). Line profiles were drawn through the center of the lumen and applied at identical positions across all reconstructions (Supplementary Fig. 1). Extracted intensity profiles were normalized for nominal intraluminal HU and 10–90% rise distance and edge width at half maximum (EWHM) were calculated at the plaque-lumen interface.

### Objective image quality measurements

Objective image quality parameters were assessed by a reader with 3 years of experience in cardiovascular imaging (S.H.) using RadiAnt DICOM Viewer (version 2025.2). To measure mean plaque attenuation (HU_plaque_), circular regions of interest (ROIs) were placed in the phantom and in vivo plaques. Phantom image noise was defined as the standard deviation (SD) of attenuation values measured in a circular ROI placed in the water tank. The mean attenuation of the same ROI in the water tank served as background attenuation (HU_background_). In vivo image noise was defined as the SD of an ROI placed in the aortic root at the level of the left main coronary ostium. Background attenuation was obtained from circular ROIs placed in the pericoronary adipose tissue adjacent to the respective plaques. All ROIs were copied to identical positions for all reconstructions. Contrast-to-noise ratio (CNR) was calculated using the following formula:

$$\:\mathrm{C}\mathrm{N}\mathrm{R}=\frac{{\mathrm{H}\mathrm{U}}_{\mathrm{p}\mathrm{l}\mathrm{a}\mathrm{q}\mathrm{u}\mathrm{e}}-{\mathrm{H}\mathrm{U}}_{\mathrm{b}\mathrm{a}\mathrm{c}\mathrm{k}\mathrm{g}\mathrm{r}\mathrm{o}\mathrm{u}\mathrm{n}\mathrm{d}}}{\mathrm{i}\mathrm{m}\mathrm{a}\mathrm{g}\mathrm{e}\:\mathrm{n}\mathrm{o}\mathrm{i}\mathrm{s}\mathrm{e}}$$

### Quantitative stenosis measurements

Phantom lumen dimensions were derived from full width at half maximum measurements based on the line profiles. In vivo PDS was calculated by the same reader, using previous methodology [4]. A commercial software (Syngo.Via, Siemens Healthineers) was used to calculate in vivo PDS by outlining the lumen at the location of maximal stenosis and proximal and distal reference segments. To ensure consistency, stenosis and reference segments were maintained identical across all reconstructions. To evaluate reproducibility, a second reader with 7 years of experience in cardiovascular imaging (M.V.N.), blinded to the initial measurements, quantified 30 stenoses. To assess inter-modality agreement with an invasive reference standard, patients who underwent invasive coronary angiography (ICA) after CCTA were identified and stenoses were semi-quantitatively assessed based on the CAD-RADS system.

### Subjective analysis

For the qualitative analysis of image noise, plaque conspicuity and diagnostic confidence, the image datasets were anonymized and read in random order by the same first reader. A four-point visual Likert scale was used for rating, with a score range from 1 (poor) to 4 (excellent) (Supplementary Table 1). The same second reader, blinded to the results of the first reader, evaluated a subset of 30 NCPs to evaluate reproducibility. Both readers were blinded to the reconstruction settings of the analyzed images.

### Statistical analysis

For statistical analysis and graphical representation, R (version 4.5.1), RStudio (2025.09.1 + 401 “Cucumberleaf Sunflower” Release), and GraphPad Prism (version 10.0.1 for Windows, GraphPad Software) were used. Continuous variables are presented as median with interquartile ranges (25th − 75th percentile). To compare objective and subjective image quality parameters between the eight reconstruction settings, the Friedman test was used. For post-hoc pairwise comparisons, the Wilcoxon signed-rank test was performed after Bonferroni correction; *p* < 0.002 was considered statistically significant. To determine inter-reader agreement for PDS values, two-way random effects intraclass correlation coefficient (ICC) was calculated [14]. Inter-reader agreement on Coronary Artery Disease-Reporting and Data System (CAD-RADS) grade and subjective scores was assessed by Gwet’s AC2 with bootstrap-derived 95% confidence intervals (CI) [15].

## Results

### Study population

Of the 275 patients screened, 217 were excluded due to the absence of non-calcified coronary plaques and 3 due to unavailable raw CT data. A total of 55 patients (age, 74 [66.5–78.0] years; 19 female and 36 male) with 97 non-calcified coronary plaques were retrospectively included in the final cohort. Demographic patient data and acquisition parameters are presented in Table 1.

**Table 1:** Patient characteristics and CT acquisition parameters

	*n* = 55
*Demographic parameters*	
Age (years)	74 (66.5–78.0)
Female sex	19 (34.5%)
BMI (kg/m^2^)	28.0 (24.7–32.3)
Hypertension	44 (80.0%)
Dyslipidemia	38 (69.1%)
Current smoking	15 (27.3%)
Diabetes	18 (32.7%)
*Acquisition parameters*	
Scan mode	
Spiral	47 (85.5%)
Sequential	8 (14.5%)
Heart rate	61 (56.5–71.0)
*Tube potential*	
90 kVp	2 (3.6%)
110 kVp	1 (1.8%)
120 kVp	44 (80.0%)
130 kVp	1 (1.8%)
140 kVp	7 (12.7%)
Dose length product (mGy·cm)	979.0 (643.0–1268.0)
Effective dose (mSv)	14.0 (9.0–17.8)

Categorical data presented as n (%); continuous data presented as median (25th–75th percentile)

BMI, Body mass index

### Edge sharpness

**Fig. 1:**
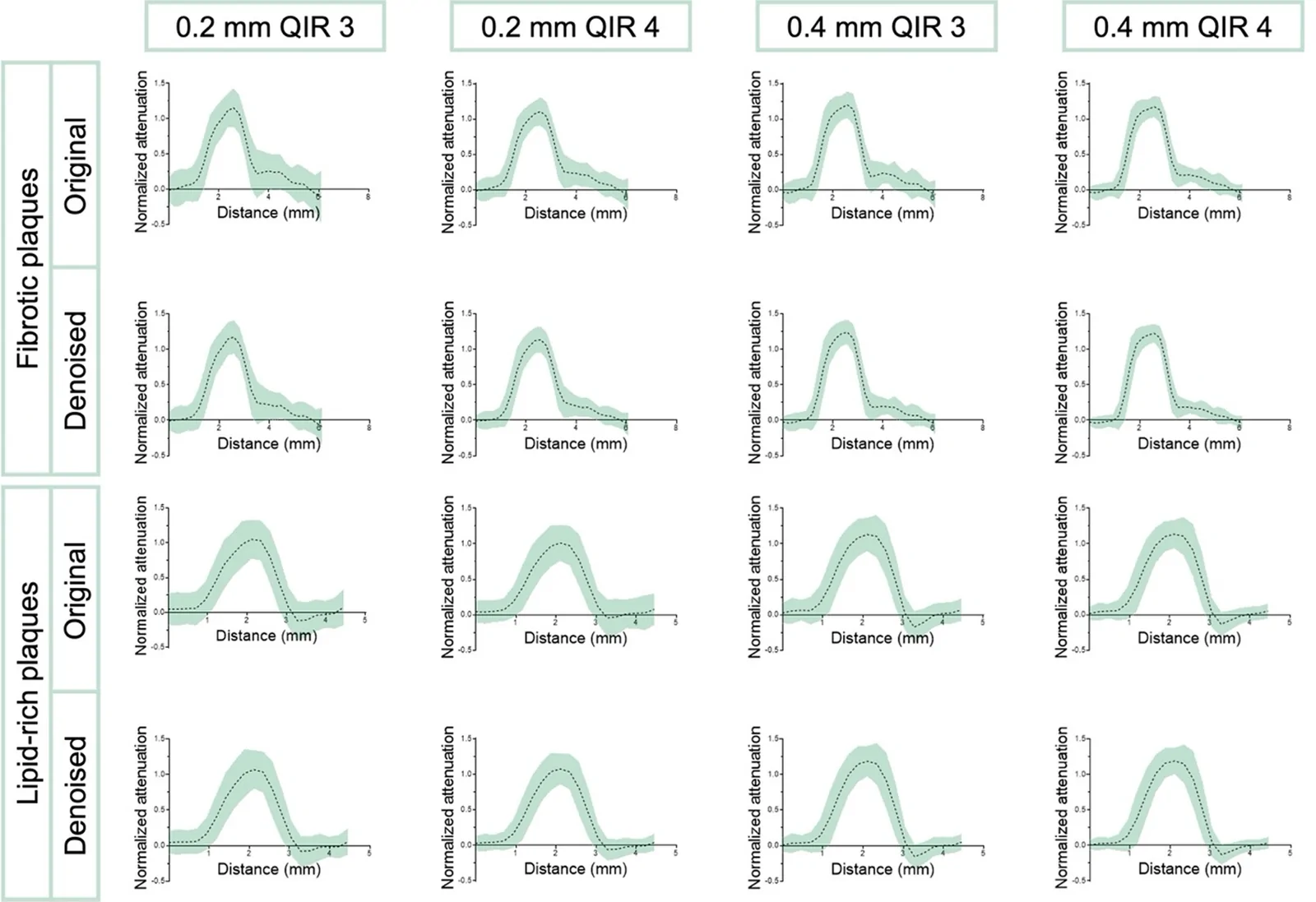
Normalized attenuation line profiles in the phantom model for fibrotic and lipid-rich plaques across different reconstruction settings, with and without convolutional neural network (CNN) denoising; QIR *=* quantum iterative reconstruction

The EWHM and the 10–90% rise distance at the lumen–plaque interfaces were not altered by denoising for either phantom plaque type (all pairwise *p* > 0.051). The coefficient of variation (CV) of EWHM and of the 10–90% rise distance decreased consistently across all reconstructions after denoising. For fibrotic plaques, the CV of EWHM decreased from 11.9 to 35.8% to 10.0–29.0% after denoising, while the CV of the 10–90% rise distance decreased from 8.5 to 12.9% to 7.1–8.6%. For lipid-rich plaques, the CV of EWHM decreased from 11.8 to 24.0% to 10.4–21.4%, and the CV of the 10–90% rise distance decreased from 5.0 to 11.7% to 5.0–10.3% following denoising. Line profile analysis revealed lower variability of attenuation values in denoised images compared to the original series (as seen in Fig. 1). All quantitative phantom measurements are summarized in Table 2.

### Objective image quality

**Fig. 2:**
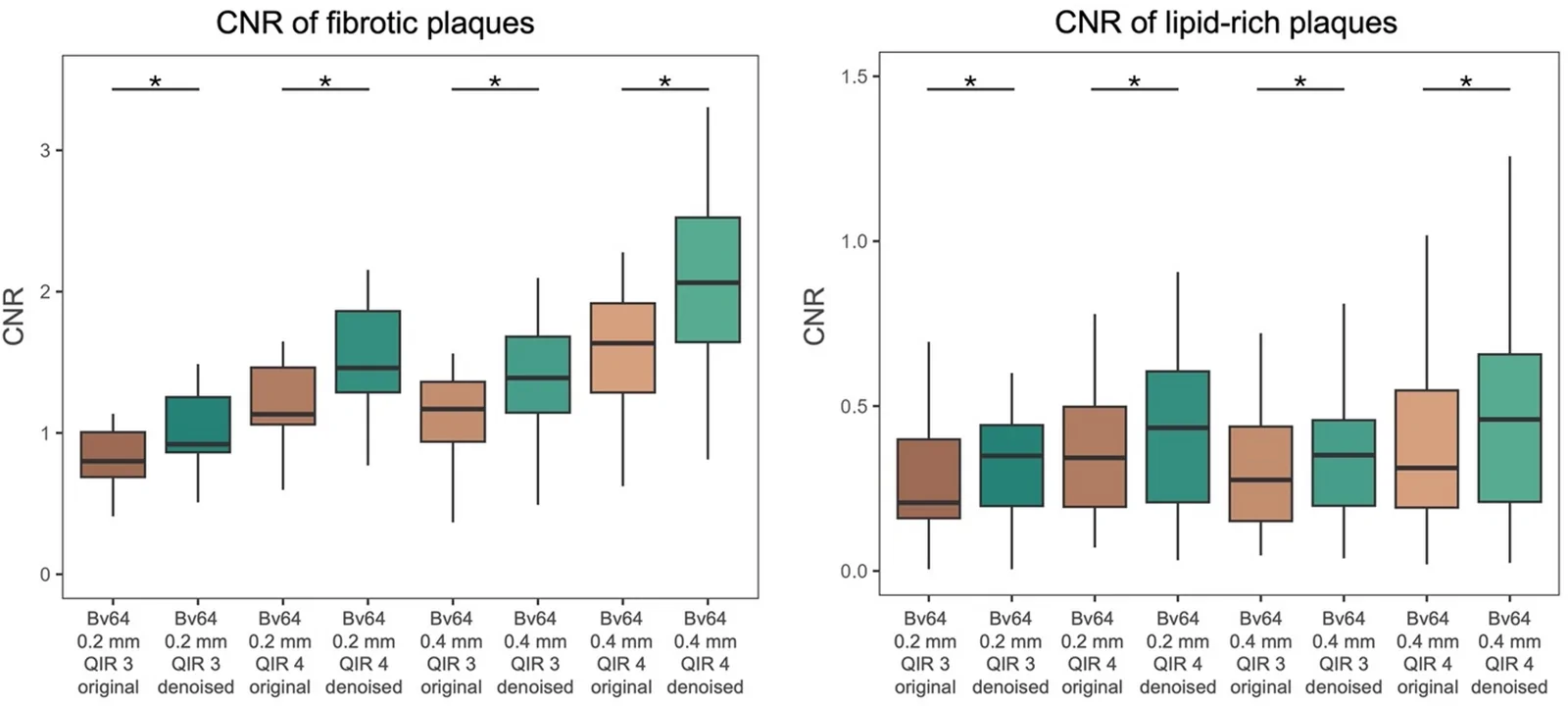
Box-and-whisker plots depicting the contrast-to-noise ratio (CNR) of the simulated fibrotic and lipid-rich plaques in the phantom model across different reconstruction settings, with (green) and without (brown) convolutional neural network (CNN) denoising; asterisks (*) indicate significant differences in pairwise comparisons; QIR = quantum iterative reconstruction

CNN denoising resulted in a consistent increase in plaque CNR across all reconstruction settings in the phantom model for both fibrotic and lipid-rich plaques (all pairwise *p* < 0.001). For fibrotic plaques, median CNR values increased from 0.80 to 1.64 in the original reconstructions to 0.92–2.06 after denoising, while for lipid-rich plaques, CNR increased from 0.21 to 0.34 to 0.35–0.46 following denoising across different slice thickness and QIR settings (Table 2). Phantom CNR values are depicted in Fig. 2.

**Fig. 3:**
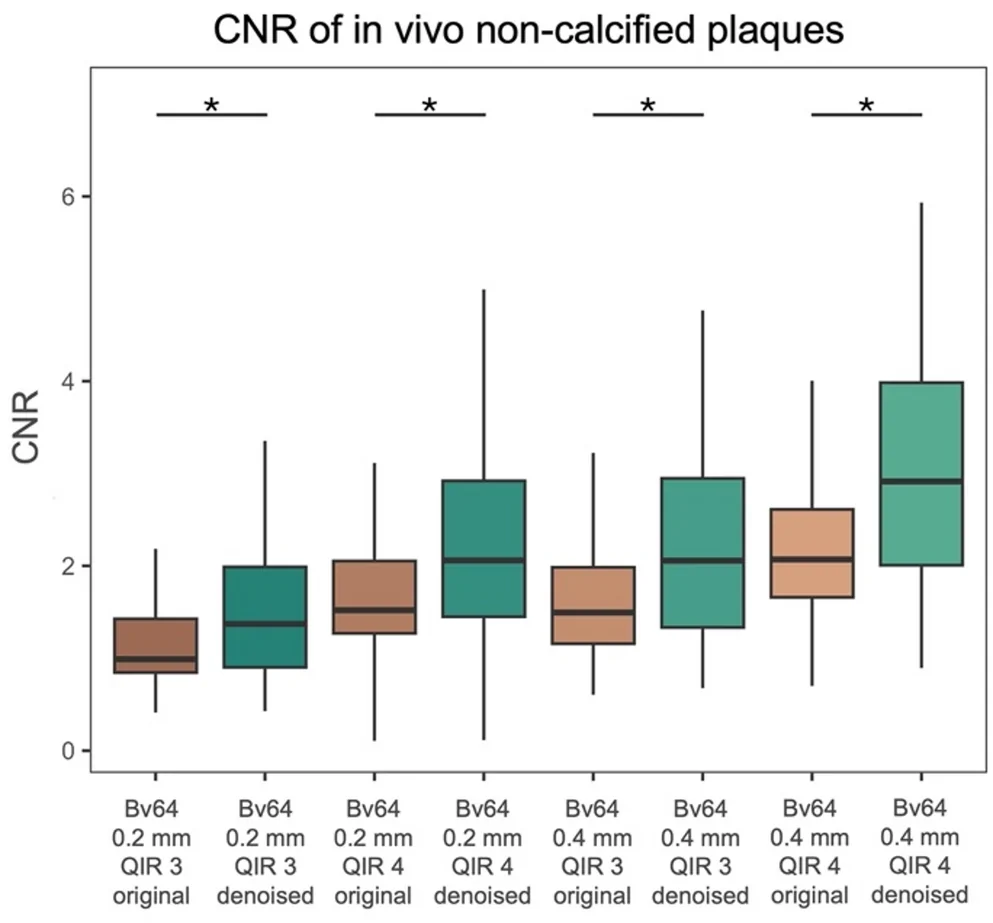
Box-and-whisker plots depicting the contrast-to-noise ratio (CNR) of the non-calcified plaques (NCPs) in vivo across different reconstruction settings, with (green) and without (brown) convolutional neural network (CNN) denoising; asterisks (*) indicate significant differences in pairwise comparisons; QIR = quantum iterative reconstruction

Similarly, CNN denoising increased in vivo CNR of NCPs across all reconstruction settings (all pairwise *p* < 0.001). Median CNR values ranged from 0.99 to 2.07 in the original reconstructions and increased to 1.37–2.92 in the denoised series. Detailed in vivo CNR values are illustrated in Fig. 3. The results of the quantitative in vivo measurements are summarized in Table 3.

### Quantitative stenosis

**Fig. 4:**
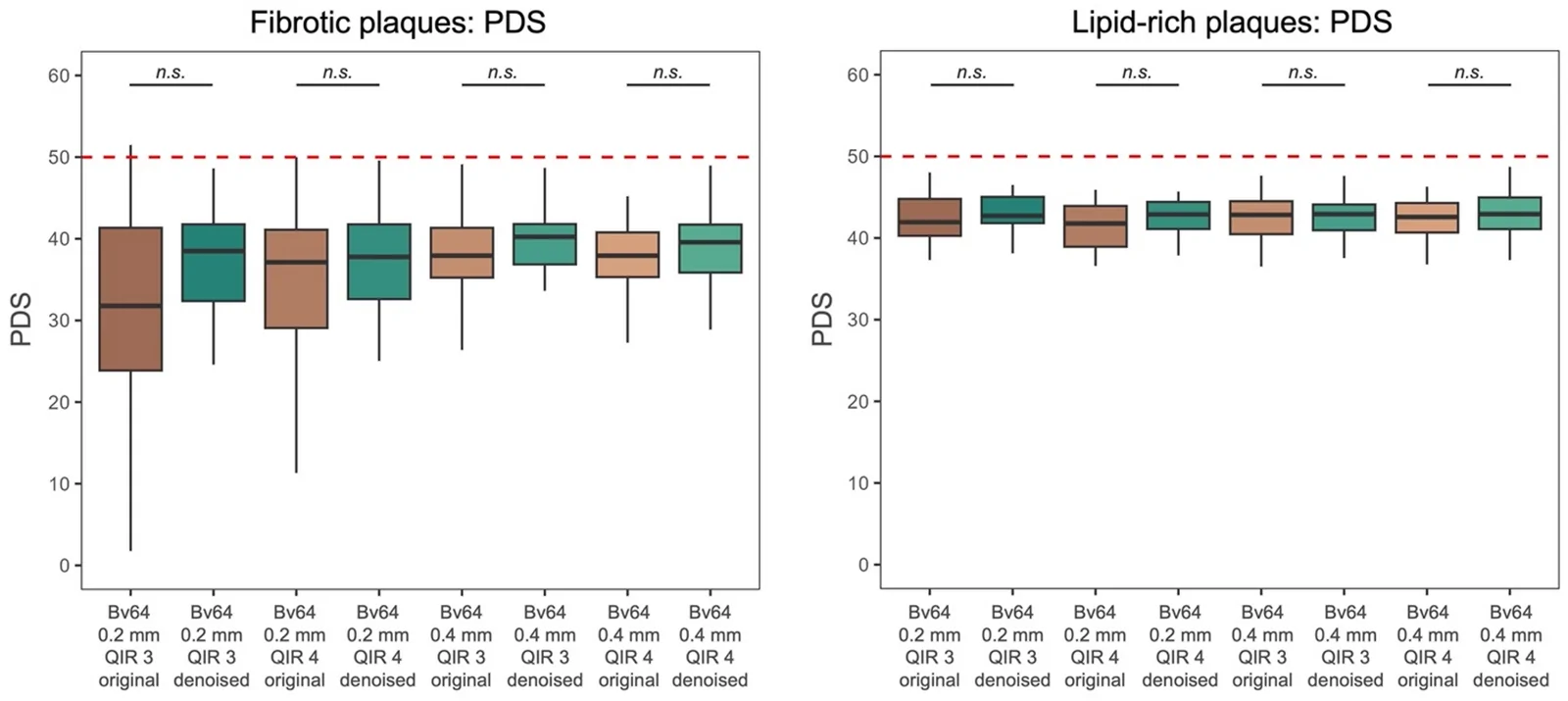
Box-and-whisker plots illustrating the measured percent diameter stenosis (PDS [%]) caused by the simulated fibrotic and lipid-rich plaques in the phantom model across different reconstruction settings, without (brown) and with convolutional neural network (CNN) denoising (green); dashed red lines at 50% represent the PDS ground truth based on phantom specifications; n.s. = not significant; QIR =quantum iterative reconstruction

CNN denoising did not alter calculated PDS for either fibrotic or lipid-rich plaques in the phantom model across reconstructions (all pairwise *p* > 0.006). In fibrotic plaques, median PDS values ranged from 31.8 to 37.9% in the original reconstructions and from 37.8 to 40.2% after denoising, while lipid-rich plaques showed median PDS values ranging from 41.8 to 42.8% before and from 42.7 to 42.9% after denoising across reconstructions. CNN denoising consistently reduced measurement variability. For fibrotic plaques, the CV of PDS decreased from 17.6 to 57.5% in the original reconstructions to 16.4–32.8% after denoising. For lipid-rich plaques, the CV of PDS decreased from 8.8 to 12.4% to 6.6–10.9% following denoising (Table 2). The distribution of phantom PDS values is shown in Fig. 4.

In vivo, CNN denoising did not affect calculated PDS values across reconstruction settings (*p* = 0.84), with median PDS values ranging from 27.0 to 28.0% in the original reconstructions and from 27.0 to 29.0% in the denoised series. Variability of in vivo PDS measurements remained comparable across reconstructions (Table 3).

In 46 patients who underwent ICA after CCTA, comprising 72 plaques, inter-modality agreement between ICA-derived and CCTA-derived equivalent stenosis categories was high, with quadratic-weighted Gwet’s AC2 values ranging from 0.81 (95% CI, 0.70–0.89) to 0.85 (95% CI, 0.68–0.91) across reconstructions. There was no evidence that CNN denoising affected agreement with ICA compared with the original reconstructions (all pairwise *p* > 0.210).

**Fig. 5:**
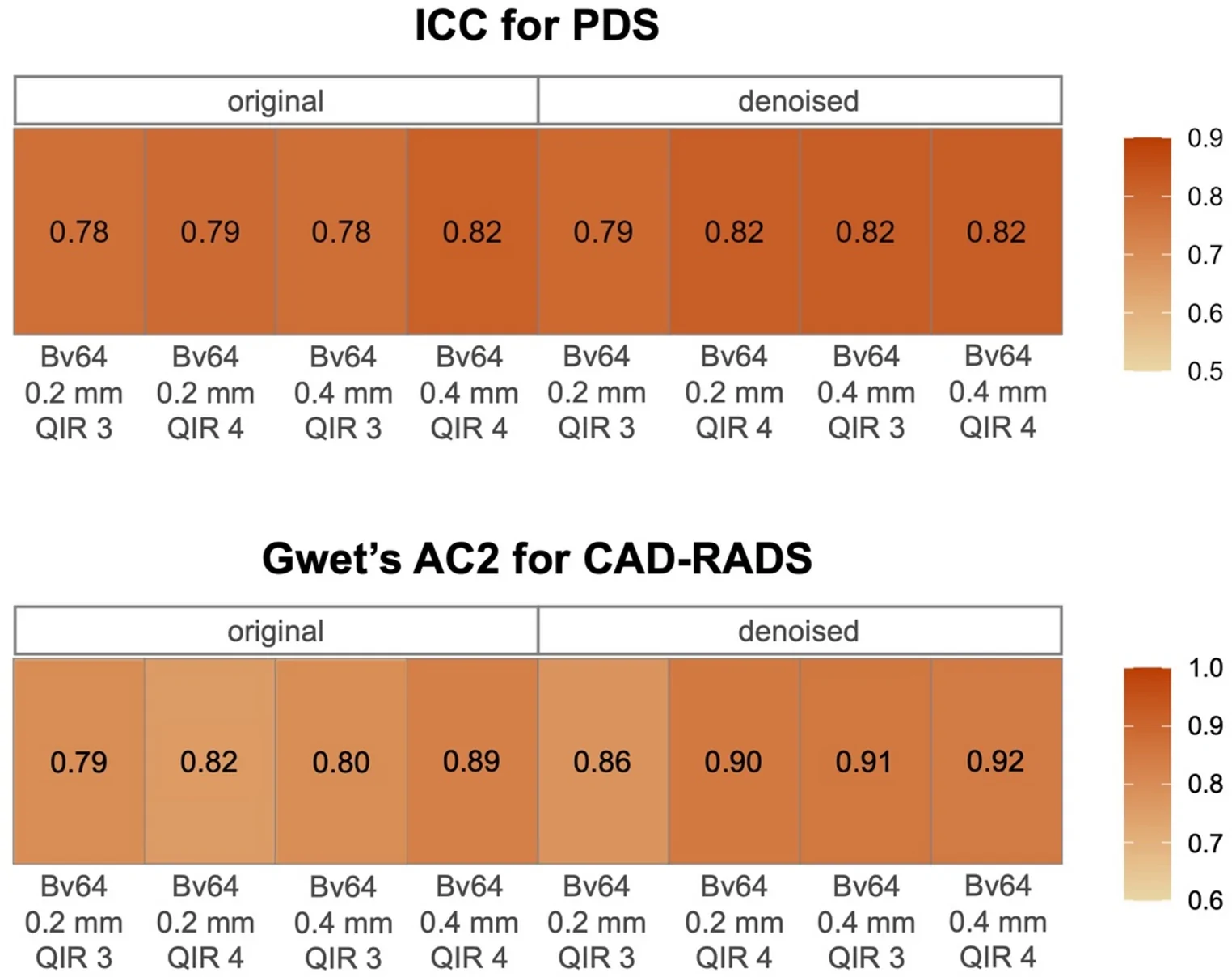
Inter-reader agreement for PDS and CAD-RADS category of the in vivo non-calcified plaques; Heat maps illustrating ICC values for PDS and quadratic-weighted Gwet’s AC2 for CAD-RADS between the two readers across the four reconstruction settings, with and without convolutional neural network (CNN) denoising; CAD-RADS = Coronary Artery Disease Reporting and Data System, ICC = intraclass correlation coefficient, PDS = percent diameter stenosis, QIR = quantum iterative reconstruction

The inter-reader agreement for PDS values was consistently good across all reconstruction settings, with ICC values ranging from 0.78 (95% CI, 0.52–0.90) to 0.82 (95% CI, 0.65–0.91) in the original reconstructions and 0.79 (95% CI, 0.56–0.90) to 0.82 (95% CI, 0.67–0.91) after CNN denoising. For inter-reader agreement with respect to CAD-RADS grading, quadratic-weighted Gwet’s AC2 values were higher after denoising: from a range between 0.79 (95% CI, 0.66–0.90) and 0.89 (95% CI, 0.81–0.95) in the original reconstructions they increased to 0.86 (95% CI, 0.75–0.94) and 0.92 (95% CI, 0.80–0.96) after denoising. Figure 5 summarizes the results of inter-reader reproducibility.

**Table 2:** Quantitative measurements in the dynamic phantom

	Bv64 0.2 mm QIR 3 original	Bv64 0.2 mm QIR 4 original	Bv64 0.4 mm QIR 3 original	Bv64 0.4 mm QIR 4 original	Bv64 0.2 mm QIR 3 denoised	Bv64 0.2 mm QIR 4 denoised	Bv64 0.4 mm QIR 3 denoised	Bv64 0.4 mm QIR 4 denoised	*p*
*Fibrotic plaques in the phantom*									
EWHM (mm)	1.41 (0.85–1.49)	1.44 (1.20–1.50)	1.38 (1.10–1.48)	1.44 (1.27–1.51)	1.40 (1.09–1.48)	1.44 (1.28–1.52)	1.39 (1.23–1.47)	1.45 (1.27–1.50)	< 0.001
CV_EWHM_ (%)	35.8	25.8	22.1	11.9	29.0	18.6	13.7	10.0	
10–90% rise distance (mm)	1.00 (0.92–1.06)	1.04 (1.01–1.10)	1.05 (0.98–1.13)	1.04 (0.98–1.11)	1.03 (1.00-1.07)	1.04 (1.03–1.09)	1.04 (0.98–1.09)	1.05 (0.99–1.09)	0.042
CV_10–90%_ (%)	12.9	8.9	10.0	8.5	8.6	7.6	6.9	7.1	
CNR	0.80 (0.69–1.01)	1.13 (1.06–1.46)	1.17 (0.94–1.36)	1.64 (1.29–1.92)	0.92* (0.86–1.25)	1.46* (1.29–1.86)	1.39* (1.14–1.68)	2.06* (1.64–2.53)	< 0.001
CV_CNR_ (%)	28.2	25.5	25.3	25.6	29.2	27.9	28.2	28.6	
PDS (%)	31.8 (23.9–41.3)	37.1 (29.1–41.1)	37.9 (35.2–41.3)	37.9 (35.3–40.8)	38.5 (32.4–41.8)	37.8 (32.6–41.8)	40.2 (36.9–41.8)	39.6 (35.9–41.7)	0.064
CV_PDS_ (%)	57.5	35.8	31.7	17.6	32.8	31.3	17.7	16.4	
*Lipid-rich plaques in the phantom*									
EWHM (mm)	1.19 (0.93–1.34)	1.27 (1.13–1.36)	1.23 (1.13–1.40)	1.30 (1.20–1.41)	1.19 (0.95–1.33)	1.28 (1.11–1.36)	1.24 (1.14–1.37)	1.31 (1.21–1.39)	< 0.001
CV_EWHM_ (%)	24.0	16.4	17.3	11.8	21.4	14.0	12.3	10.4	
10–90% rise distance (mm)	0.95 (0.90–0.99)	0.97 (0.92-1.00)	0.93 (0.89–0.99)	0.94 (0.91–0.97)	0.96 (0.93–0.99)	0.96 (0.93–0.99)	0.92 (0.90–0.98)	0.94 (0.9–0.99)	0.003
CV_10–90%_ (%)	11.7	5.0	10.4	6.5	10.3	5.0	6.5	5.8	
CNR	0.21 (0.160–0.399)	0.34 (0.194–0.498)	0.28 (0.151–0.438)	0.31 (0.192–0.547)	0.35* (0.197–0.442)	0.43* (0.208–0.605)	0.35* (0.198–0.457)	0.46* (0.209–0.657)	< 0.001
CV_CNR_ (%)	67.7	73.1	71.3	84.2	68.6	79.0	77.3	88.8	
PDS (%)	42.0 (40.3–44.8)	41.8 (38.9–43.9)	42.8 (40.5–44.5)	42.6 (40.7–44.3)	42.7 (41.8–45.0)	42.9 (41.1–44.4)	42.9 (41.0-44.1)	42.9 (41.1–45.0)	0.024
CV_PDS_ (%)	11.5	8.8	12.4	9.6	10.9	6.6	8.3	9.4	

Data presented as median (25th–75th percentile). Friedman test was used to compare reconstruction settings, using post-hoc Wilcoxon signed-rank test with a Bonferroni corrected *p* < 0.002 for pairwise comparisons

Asterisks (*) indicate a significant difference between denoised and original images

CNR, contrast-to-noise ratio; CV, coefficient of variation; EWHM, Edge width at half maximum; PDS, percent diameter stenosis; QIR, quantum iterative reconstruction

**Table 3:** Quantitative measurements in vivo

	Bv64 0.2 mm QIR 3 original	Bv64 0.2 mm QIR 4 original	Bv64 0.4 mm QIR 3 original	Bv64 0.4 mm QIR 4 original	Bv64 0.2 mm QIR 3 denoised	Bv64 0.2 mm QIR 4 denoised	Bv64 0.4 mm QIR 3 denoised	Bv64 0.4 mm QIR 4 denoised	*P*
CNR	0.99 (0.85–1.43)	1.52 (1.27–2.05)	1.50 (1.16–1.99)	2.07 (1.66–2.61)	1.37* (0.90–1.99)	2.06* (1.45–2.92)	2.06* (1.33–2.95)	2.92* (2.01–3.98)	< 0.001
CV_CNR_ (%)	36.2	36.0	38.7	33.0	46.1	46.3	53.7	42.5	
PDS (%)	28.0 (17.8–39.8)	27.0 (17.0-38.3)	28.0 (15.8–41.0)	27.5 (17.8–41.0)	27.5 (19.0–39.0)	28.0 (17.8–39.8)	27.0 (19.0–40.0)	29.0 (17.0–39.0)	0.84
CV_PDS_ (%)	59.6	59.7	57.2	56.9	56.1	57.3	56.6	54.9	
Gwet’s AC2	0.83 [0.70–0.90]	0.81 [0.70–0.89]	0.85 [0.68–0.91]	0.83 [0.70–0.90]	0.82 [0.72–0.90]	0.84 [0.74–0.91]	0.84 [0.72–0.91]	0.85 [0.67–0.91]	N/A

Data presented as median (25th–75th percentile). Friedman test was used to compare reconstruction settings, using post-hoc Wilcoxon signed-rank test with a Bonferroni corrected *p* < 0.002 for pairwise comparisons. Gwet’s AC2 values represent quadratic-weighted intermodality agreement between CCTA-derived CAD-RADS stenosis categories and ICA-derived stenosis categories; values are shown with 95% confidence intervals

Asterisks (*) indicate a significant difference between denoised and original images

*CNR*,* contrast-to-noise ratio; CV*,* coefficient of variation; PDS*,* percent diameter stenosis; QIR*,* quantum iterative reconstruction.*

### Subjective analysis

**Fig. 6:**
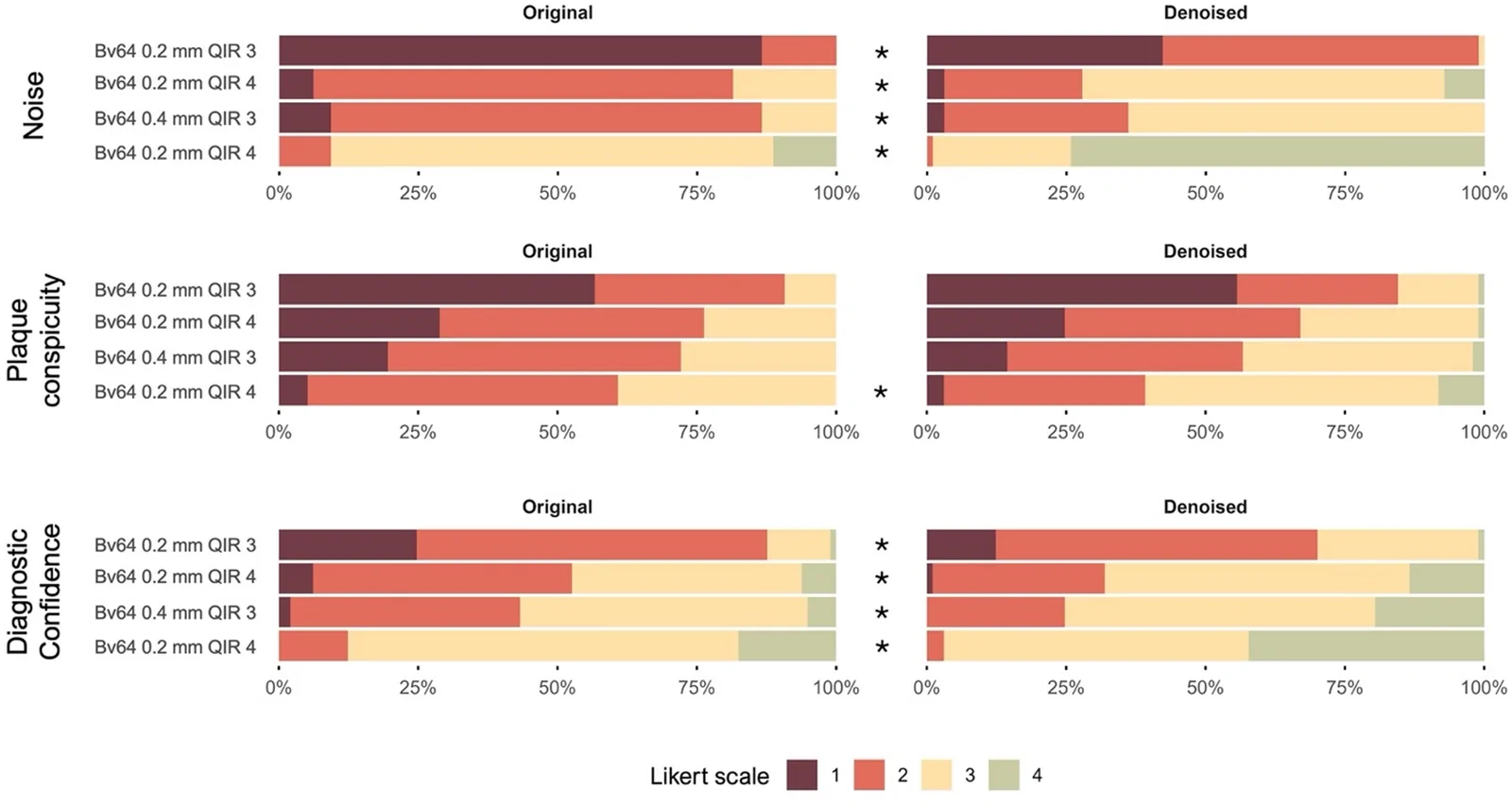
Subjective image quality analysis of image noise, plaque conspicuity and diagnostic confidence of in vivo non-calcified plaques, with (right) and without (left) convolutional neural network (CNN) denoising; asterisks (*) indicate significant differences in pairwise comparisons; QIR = quantum iterative reconstruction

Subjective image quality assessment of non-calcified plaques demonstrated a consistent improvement in perceived noise following CNN denoising (all pairwise *p* < 0.001). While CNN denoising did not alter subjective plaque conspicuity in three of the four reconstruction settings (all pairwise *p* < 0.002), it improved conspicuity for Bv64 0.4 mm QIR 4 (2.0 [2.0–3.0] vs. 3.0 [2.0–3.0]; *p* < 0.001). Diagnostic confidence improved consistently following CNN denoising across all reconstruction settings (all pairwise *p* < 0.001). Gwet’s AC2 values calculated for interreader agreement were as follows: for image noise, 0.90 (95% CI, 0.86–0.94) in the original and 0.90 (95% CI, 0.87–0.94) in the denoised series; for plaque conspicuity/sharpness, 0.77 (95% CI, 0.64–0.85) in the original and 0.66 (95% CI, 0.51–0.78) in the denoised series; and for diagnostic confidence, 0.79 (95% CI, 0.72–0.85) in the original and 0.78 (95% CI, 0.60–0.89) in the denoised series. Figure 6 illustrates the detailed results of the subjective image quality analysis.

## Discussion

The present combined phantom and in vivo study evaluated the added value of post-hoc CNN denoising for the assessment of non-calcified coronary plaques on UHR PCD-CCTA series. With the use of CNN denoising, the main findings are as follows: (1) Sharpness at the lumen–plaque interface was preserved, with a reduction in measurement variability across acquisitions; (2) Plaque CNR increased across all reconstruction settings, without altering PDS values; and (3) Subjective image quality improved with respect to noise and diagnostic confidence.

The UHR visualization of NCPs is currently disadvantaged in clinical routine by calcification-oriented reconstruction settings and poorer performance of iterative reconstruction in low-attenuation lesions [3, 7, 8]. We demonstrate that CNN denoising can mitigate the inherent noise penalty of UHR reconstructions while preserving lumen–plaque sharpness, allowing the spatial advantages of the thinnest slices to be retained for NCP visualization. Notably, denoising increased the CNR of 0.2 mm reconstructions to levels comparable to the corresponding 0.4 mm series, effectively combining the anatomical detail of the thinnest slices with the lower image noise associated with doubled z-axis thickness [16]. The reduction in measurement variability indicates that the algorithm improves the stability of plaque–lumen interface depiction rather than inducing over-smoothing, a conclusion supported by the absence of changes in PDS values. Finally, the improvements in subjective evaluation indicate that the impact of denoising extends beyond isolated numerical metrics and is visually apparent, while the observed improvement in CAD-RADS agreement demonstrates that these image-level effects translate into more consistent interpretive decisions. Of note, the systematic underestimation of phantom PDS relative to the nominal 50% stenosis is likely methodological and reflects the inherent limitations of manual line-profile placement. Even with careful positioning, profiles rarely intersect the exact geometric center of the lumen, preventing precise 50% diameter measurements and resulting in slightly lower calculated PDS values.

The CNN-based denoising algorithm used in this study has previously been investigated in CCTA on EID-CT. Hong et al. demonstrated significant CNR increases of intraluminal coronary attenuation following denoising, comparable in magnitude to the relative gains observed in our work [9]. Lanzafame et al. also reported CNR improvements for intraluminal coronary attenuation on EID-CT, with even larger increases when applying maximal denoising strength [10]. Regarding sharpness, Hong et al. observed shorter edge-rise distances at the vessel wall after denoising, whereas in our study, lumen–plaque sharpness metrics remained unchanged [9]. Together, these findings support that the algorithm does not compromise spatial resolution and anatomical detail, which is a prerequisite for reliable assessment of NCPs. Prior studies applying this algorithm have primarily evaluated diagnostic accuracy using binary stenosis quantification [9, 10], whereas, to our knowledge, no previous investigation has systematically quantified PDS across different NCP types, under static and dynamic conditions, across multiple reconstruction settings, nor examined reproducibility in vivo. Our results extend the existing literature by expanding previous observations to PCD-CT technology, demonstrating unchanged PDS across all evaluated conditions and showing that, through improvements in inter-reader agreement, denoising can enhance interpretive consistency in the non-calcified plaque domain of UHR PCD-CT.

The present findings indicate that CNN denoising could support the routine use of thin UHR reconstructions for NCP analysis, without the need to resort to thicker slices or softer kernels solely to manage noise. Improved robustness and reproducibility of stenosis reporting could enable more consistent risk stratification and downstream management decisions, while higher diagnostic confidence suggests that plaque interpretation may be carried out with greater certainty, reducing equivocal reads and minimizing variability in clinical recommendations. Furthermore, more noise-controlled and measurement-stable UHR series may also improve the feasibility of longitudinal plaque monitoring and strengthen the use of quantitative plaque metrics and AI-assisted analysis.

The following limitations should be acknowledged. First, the single-center, retrospective design with a moderate number of patients and NCPs may limit generalizability. Second, given the plaque-level, multi-reconstruction study design and time-intensive analysis, interreader reproducibility for subjective image quality and quantitative stenosis measurements was assessed in a randomly selected subset comprising 31% (30/97) of the plaques. While this approach allowed reproducibility to be assessed across all 8 reconstruction settings, it does not replace a fully duplicated independent analysis of the entire cohort. Third, we evaluated only one commercially available denoising algorithm and only at a single denoising strength. Future studies could assess further denoising levels to quantify potential over-smoothing and its effect on edge sharpness. Fourth, in contrast to the phantom, non-calcified plaques in vivo were not subcategorized into lipid-rich and fibrotic. Fifth, although this study focused on NCPs, we did not assess the effect of denoising on calcified plaques. Such work is necessary to determine whether denoised UHR series could serve as a one-stop dataset for all plaque types. Sixth, despite blinding, subjective image quality assessment may still have been influenced by recognizable differences in noise texture between series. Seventh, we did not investigate whether CNN denoising improves detection of subtle NCPs or quantitative characterization of high-risk plaque features. Therefore, the observed CNR improvement should only be interpreted as an image-quality finding; dedicated diagnostic accuracy studies are needed to determine whether denoising translates into improved detection or characterization of NCPs in clinical practice.

In summary, CNN denoising improves UHR PCD-CCTA visualization of non-calcified coronary plaques by increasing plaque CNR and diagnostic confidence while preserving sharpness and stenosis measurement consistency. These findings support its potential integration into UHR clinical workflows to enable reliable thin-slice CCTA assessment for NCPs as well.

## Supplementary Information

Below is the link to the electronic supplementary material.

Supplementary Material 1

## Data Availability

The data supporting the results of this study cannot be made publicly available due to sensitive patient-level clinical and imaging information. Access may be granted by the corresponding author upon reasonable request, subject to applicable ethical and data protection requirements.

## Abbreviations

CAD-RADS: Coronary Artery Disease Reporting and Data System
CCTA: Coronary CT angiography
CNN: Convolutional neural network
CNR: Contrast-to-noise ratio
CV: Coefficient of variation
EID: Energy-integrating detector
EWHM: Edge width at half maximum
HU: Hounsfield unit
ICC: Intraclass correlation coefficient
NCP: Non-calcified plaque
PCD: Photon-counting detector
PDS: Percent diameter stenosis
QIR: Quantum iterative reconstruction
ROI: Region of interest
SD: Standard deviation
UHR: Ultrahigh-resolution
