# Supplementary material for: Deep-learning denoising for ultrahigh-resolution photon-counting detector CT: phantom and in vivo evaluation of non-calcified coronary plaques
Source: Int J Cardiovasc Imaging. 2026 Jul 10;42(11):2085–95. doi: 10.1007/s10554-026-03774-x (PMC13745421; doi:10.1007/s10554-026-03774-x)
Supplement: Supplementary file 1 [file 10554_2026_3774_MOESM1_ESM.docx]

**SUPPLEMENTAL MATERIAL**

| **Table S1.** Likert scores for noise, plaque conspicuity and diagnostic confidence | | | |
| --- | --- | --- | --- |
| **Likert score** | **Noise** | **Plaque conspicuity** | **Diagnostic confidence** |
| 1 | Pronounced graininess, impairing plaque visualization | Very blurry: poorly defined plaque-lumen interface | Non-diagnostic: unusable for diagnosis |
| 2 | Noticeable graininess, plaque still identifiable | Blurry, but visible plaque-lumen interface | Reduced: can only differentiate between obstructive vs. non-obstructive stenoses |
| 3 | Mild noise, clear plaque visualization | Mild blurring, but clear delineation of the plaque-lumen interface | Good: assignable CAD-RADS score despite artifacts/noise |
| 4 | Excellent plaque visualization | Sharp: well-defined plaque-lumen interface | Excellent: very high confidence in diagnosis |
| *^CAD-RADS,^* *^Coronary Artery Disease Reporting and Data System.^* | | | |

**Fig. S1 –** Line profile analysis for the calculation of plaque edge sharpness in the coronary phantom. Coronary tubes are filled with contrast material yielding intraluminal attenuations of 300, 400, or 500 Hounsfield units (HU). Ultrahigh-resolution images reconstructed with 0.2 mm and 0.4 mm slice thickness and quantum iterative reconstruction (QIR) levels 3 and 4 are shown for the original (top row) and convolutional neural network (CNN) denoised datasets (second row). The yellow line indicates the linear region of interest drawn perpendicular to the lumen–plaque interface and used to extract attenuation profiles. The corresponding line profiles for the original (third row) and CNN denoised (bottom row) images were used to derive quantitative sharpness metrics (10–90% rise distance and edge width at half maximum).
